# Soil microbiome of shiro reveals the symbiotic relationship between *Tricholoma bakamatsutake* and *Quercus mongolica*

**DOI:** 10.3389/fmicb.2024.1361117

**Published:** 2024-03-27

**Authors:** Hongbo Guo, Weiye Liu, Yuqi Xie, Zhenyu Wang, Chentong Huang, Jingfang Yi, Zhaoqian Yang, Jiachen Zhao, Xiaodan Yu, Lidiya Alekseevna Sibirina

**Affiliations:** ^1^College of Life Engineering, Shenyang Institute of Technology, Fushun, China; ^2^Primorye State Agricultural Academy, Ussuriysk, Russia; ^3^College of Biological Science and Technology, Shenyang Agricultural University, Shenyang, China; ^4^Federal Scientific Center of the East Asia Terrestrial Biodiversity Far Eastern Branch of Russian Academy of Sciences, Vladivostok, Russia

**Keywords:** shiro soil, ectomycorrhizal fungi, *Quercus mongolica*, rhizosphere microorganism, symbiotic system, *Tricholoma bakamatsutake*

## Abstract

*Tricholoma bakamatsutake* is a delicious and nutritious ectomycorrhizal fungus. However, its cultivation is hindered owing to limited studies on its symbiotic relationships. The symbiotic relationship between *T. bakamatsutake* and its host is closely related to the shiro, a complex network composed of mycelium, mycorrhizal roots, and surrounding soil. To explore the symbiotic relationship between *T. bakamatsutake* and its host, soil samples were collected from *T. bakamatsutake* shiro (Tb) and corresponding *Q. mongolica* rhizosphere (CK) in four cities in Liaoning Province, China. The physicochemical properties of all the soil samples were then analyzed, along with the composition and function of the fungal and bacterial communities. The results revealed a significant increase in total potassium, available nitrogen, and sand in Tb soil compared to those in CK soil, while there was a significant decrease in pH, total nitrogen, total phosphorus, available phosphorus, and silt. The fungal community diversity in shiro was diminished, and *T. bakamatsutake* altered the community structure of its shiro by suppressing other fungi, such as *Russula* (ectomycorrhizal fungus) and *Penicillium* (phytopathogenic fungus). The bacterial community diversity in shiro increased, with the aggregation of mycorrhizal-helper bacteria, such as *Paenibacillus* and *Bacillus*, and plant growth-promoting bacteria, such as *Solirubrobacter* and *Streptomyces*, facilitated by *T. bakamatsutake*. Microbial functional predictions revealed a significant increase in pathways associated with sugar and fat catabolism within the fungal and bacterial communities of shiro. The relative genetic abundance of carboxylesterase and gibberellin 2-beta-dioxygenase in the fungal community was significantly increased, which suggested a potential symbiotic relationship between *T. bakamatsutake* and *Q. mongolica*. These findings elucidate the microbial community and relevant symbiotic environment to better understand the relationship between *T. bakamatsutake* and *Q. mongolica*.

## 1 Introduction

*Tricholoma bakamatsutake*, an edible mushroom, often forms ectomycorrhizae with the roots of Fagaceae trees, such as *Quercus mongolica* (Deng, 2003; Ota et al., 2012). *T. bakamatsutake* exhibits similar morphological characteristics, aroma profiles, and nutritional composition to those of *T. matsutake* (Quan et al., 2005, 2007); however, it is priced lower in the market compared to *T. matsutake* (Kang et al., 2018). The growing demand for *T. bakamatsutake* necessitates its cultivation due to limited natural sources (Deng, 2019; Hou, 2020). Consequently, researchers are investigating optimal environmental conditions for the laboratory culture of *T. bakamatsutake* mycelia (Terashima, 1994, 1999; Wu et al., 2009; Yamanaka et al., 2019), while also attempting fruiting body production through transplantation into natural habitats (Kawai et al., 2018; Hou, 2020). However, disparities between natural and laboratory environments exist, and factors like soil physicochemical properties and microbiome composition may influence fruiting body yield in natural environments.

The underground mycelia of *T. bakamatsutake* form a white, sponge-like structure called shiro (Ogawa, 1977). In *T. matsutake*, the shiro plays an important role in the formation of fruiting bodies (Yun et al., 1997), which form at the core of the shiro. High-throughput sequencing has been widely used to study the microbiome in the shiro (Kim et al., 2014; Oh et al., 2016; Ye et al., 2018), allowing for the discovery of microbial diversity and non-culturable microorganisms by directly analyzing genetic material from shiro samples (Shokralla et al., 2012; Kim et al., 2014). Kim et al. (2014) investigated bacterial communities in *T. matsutake* shiro using pyrophosphate sequencing and found that environmental factors may have a greater influence on these communities than the dominance of *T. matsutake*. Oh et al. (2016) identified several microorganisms associated with *T. matsutake*, including *Bacillus* and *Umbelopsis*, which may benefit *T. matsutake* growth. Vaario et al. (2011) found that *T. matsutake* coexists with various fungal and actinobacterial species, and high enzymatic activity is involved in organic carbon degradation in the shiro. This approach can be used to study the symbiotic relationship between *T. bakamatsutake* and *Quercus mongolica* to provide a deeper understanding of *T. bakamatsutake* shiro and lay a foundation for future artificial cultivation. Additionally, *T. bakamatsutake* adapts more easily to the environment than *T. matsutake*, making it suitable for artificial cultivation (Deng, 2019; Hou, 2020). However, limited research on *T. bakamatsutake* shiro hinders its artificial cultivation and economic prospects.

Given its ectomycorrhizal (ECM) nature, the effects of *T. bakamatsutake* on other microorganisms, ecological milieu, and host plants necessitate careful consideration. ECM fungi colonize plant roots, modify the soil microbiome structure (Calvaruso et al., 2007; Li et al., 2019), and continuously spread their mycelia. The dominance of ECM fungi is driven by ecological niche competition, metabolite secretion, and the enrichment of mycorrhization helper bacteria (MHB) (Poole et al., 2001; Moeller and Peay, 2016). The symbiotic relationship between ECM fungi and host plants is important as the fungal mycelia closely adhere to plant roots, providing effective protection against pathogens for woody plants (Tedersoo et al., 2020). This symbiotic relationship also enhances nutrient exchange between ECM fungi and the plants; photosynthates are transported from the plants to the roots where ECM fungi reside, while minerals are transported from ECM fungi to the plants via mycorrhiza (Nehls et al., 2010; Nehls and Plassard, 2018). Additionally, ECM fungi play an important role in increasing plant resilience and improving forest environments (Song et al., 2015; Bennett et al., 2017; Yu and Yuan, 2023).

This study aimed to compare the soils of shiro and *Q. mongolica* rhizosphere to reveal the ecological relationship between *T. bakamatsutake* and *Q. mongolica* in natural forests. Further, the contributions of *T. bakamatsutake* to the host and environment were evaluated. The results herein contribute to a better understanding of the relationship between ECM fungi and their hosts in the micro-ecosystem, while providing a theoretical basis for the artificial cultivation of *T. bakamatsutake*.

## 2 Materials and methods

### 2.1 Soil sampling and determination of soil physicochemical properties

In August 2022, 28 soil samples were collected from four cities in Liaoning Province, China, Anshan (AS), Kuandian (KD), Xinbin (XB), and Xiuyan (XY). The sampling sites were situated in a mixed forest comprising *Q. mongolica* and *Lespedeza bicolor*, with *Carex callitrichos* being the predominant herbaceous plant. All sampling locations were maintained at a minimum distance of 10 m from *L. bicolor*. These 28 samples included 14 *T. bakamatsutake* shiro soil (Tb) and 14 corresponding *Q. mongolica* rhizosphere soil (CK) samples. The Tb samples were collected from the shiro soil under the fruiting body of *T. bakamatsutake.* The CK soil samples were collected from the non-mycorrhizal soil of the oak rhizosphere, located 5 m away from the shiro, ensuring that both the CK and Tb samples originated from the same oak rhizosphere. Additionally, this distance not only mitigated potential impacts of shiro on the CK samples but also minimized environmental disparities between Tb and CK. All the samples were collected by first removing the surface litter layer, then collecting soil at a depth of 5–15 cm and diameter of 10 cm using a soil sampler. The samples were encoded in the format of city name followed by Tb/CK (e.g., ASTb represented the Tb soil from Anshan). Grouping and numbering details for all samples are provided in Table 1. The samples were stored at −4°C upon the removal of the gravel and roots and subsequently divided into three parts. The first was dried and passed through a 20-mesh sieve to determine the chemical properties; pH, total nitrogen (TN), total potassium (TK), total phosphorus (TP), available nitrogen (AN), available potassium (AK), available phosphorus (AP), and organic matter (OM) were measured using previous methods (Bao, 2000). The second sample was desiccated and subsequently sieved through a 100-mesh sieve to determine its mechanical composition using the Bouyoucos hydrometer method (Bouyoucos, 1936). The third was stored at −80°C for DNA extraction.

**Table 1:** Collection and grouping of the 28 soil samples.

Cities	*T. bakamatsutake* shiro soils	*Q. mongolica* rhizosphere soils
Anshan (AS)	ASTb1, ASTb2, ASTb3	ASCK1, ASCK2, ASCK3
Kuandian (KD)	KDTb1, KDTb2, KDTb3	KDCK1, KDCK2, KDCK3
Xinbin (XB)	XBTb1, XBTb2, XBTb3, XBTb4, XBTb5	XBCK1, XBCK2, XBCK3, XBCK4, XBCK5
Xiuyan(XY)	XYTb1, XYTb2, XYTb3	XYCK1, XYCK2, XYCK3

Tb, *T. bakamatsutake* shiro soil; CK, *Q. mongolica* rhizosphere soil; AS, Anshan (123°9′26″E, 41°0′13″N, 267 m); KD, Kuandian (124°53′0″E, 41°30′15″N, 323 m); XB, Xinbin (125°24′5″E, 41°36′54″N, 662.94 m); XY, Xiuyan (123°41′35″E, 40°19′13″N, 254.53 m).

### 2.2 DNA extraction, polymerase chain reaction (PCR) amplification, and Illumina MiSeq sequencing

Total genomic DNA of the microbiome was extracted using the E.Z.N.A.^®^ soil DNA kit (Omega Bio-tek, Norcross, GA, United States) according to the manufacturer’s instructions. The quality of the extracted genomic DNA was determined using 1% agarose gel electrophoresis with a DYY-6C electrophoresis apparatus (Liuyi Biology, Beijing, China). The concentration and purity of the DNA were determined using a NanoDrop 2000 (Thermo Fisher Scientific, Waltham, MA, United States).

For bacteria, the 16S-specific primers 338F (5′-ACTCCTACGGGAGGCAGCAG-3′) and 806R (5′-GGACTACHVGGGTWTCTAAT-3′) were used to amplify different regions of the 16S ribosomal RNA (rRNA) gene. For fungi, specific primers, including the internal transcribed spacer ITS1F (5′-CTTGGTCATTTAGAGGAAGTAA-3′) and ITS2R (5′-GCTGCGTTCTTCATCGATGC-3′), were used to amplify different regions of the ITS gene. The PCR mixture included 4 μL of 5 × Fast Pfu buffer, 2 μL of 2.5 mM dNTPs, 0.8 μL of each primer (5 μM), 0.4 μL of Fast Pfu polymerase (TransGen Biotech, Beijing, China), 10 ng of template DNA, and ddH_2_O to a final volume of 20 μL. The cycling conditions were as follows: initial denaturation at 95°C for 3 min, followed by 27 cycles of denaturation at 95°C for 30 s, annealing at 55°C for 30 s, extension at 72°C for 45 s, single extension at 72°C for 10 min, and 4°C. The amplification of all the samples was performed in triplicate using the ABI GeneAmp^®^ 9,700 instrument (ABI, CA, United States). The PCR products were extracted using a 2% agarose gel and purified with the AxyPrep DNA Gel Extraction Kit (Axygen Biosciences, Union City, CA, United States) according to the manufacturer’s instructions. The purified PCR products were then quantified using a Quantus™ Fluorometer (Promega, Madison, WI, United States).

The purified PCR amplicons were pooled in equimolar amounts, paired-end, and sequenced on an Illumina MiSeq PE300 platform (Illumina, San Diego, CA, United States) according to the Majorbio Bio-Pharm Technology Co., Ltd. (Shanghai, China) protocol. Raw sequencing reads were deposited in the database of the National Center for Biotechnology Information Sequence Read Archive (BioProject: PRJNA955660).

### 2.3 Data processing

Raw FASTQ files were de-multiplexed using an in-house perl script, quality-filtered using fastp version 0.19.6 (Chen et al., 2018), and merged using FLASH version 1.2.7 (Magoc and Salzberg, 2011). The optimized sequences were clustered into operational taxonomic units (OTUs) using UPARSE 7.1 (Stackebrandt and Goebel, 1994; Edgar, 2013), with a 97% sequence similarity level. The most abundant sequence in each OTU was selected as the representative sequence. The taxonomy of each OTU representative sequence was analyzed using RDP Classifier version 2.2 (Wang et al., 2007) against the Silva 16S rRNA and Unite ITS gene databases, with a confidence threshold of 0.7. Finally, metagenomic functions were predicted for the bacterial and fungal communities. The functions of the microbial community were predicted using Phylogenetic Investigation of Communities by Reconstruction of Unobserved States (PICRUSt2), BugBase, FAPROTAX, and FunGuid, based on the OTU representative sequences. PICRUSt2^1^ is a software comprising the following series of tools: HMMER is used to align OTU representative sequences with reference sequences; PA-NG and Gappa are used to place OTU representative sequences into a reference tree; castor is used to normalize gene copies; and MinPath is used to predict gene family profiles and allocate them into gene pathways. BugBase is a tool for predicting the 16S phenotypic functionality of microbiota and can be used as a web application.^2^ For the prediction and visualization of microbial phenotypes, OTU tables and mapping files should be inputted into the BugBase operating program. FAPROTAX is the ecological function database of prokaryotes and classifies the ecological roles of bacteria and archaea in the environment according to published literature. The collapse_table.py command in FAPROTAX can be employed to assign and summarize the functional groups of bacteria into OTU taxonomic tables (Louca et al., 2016). FunGuid^3^ is the ecological function database of fungi and was constructed based on existing literature. By uploading and analyzing the fungal relative OTU taxonomic table in FunGuid, predictive results can be obtained for the ecological functionality of the fungal community.

### 2.4 Statistical analysis

Data analyzes were performed using the Majorbio Cloud platform^4^ and R (v4.0.2). Mothur (Schloss et al., 2009) software^5^ was used to calculate the alpha diversity indices, such as the Chao and Shannon’s indices. The Wilcoxon rank-sum test was used for the inter-group variation analysis of the alpha diversity indices, and a non-metric multidimensional scaling (NMDS) analysis based on the Bray-Curtis distance algorithm was used to test the similarity between the samples’ microbial community structure. The PERMANOVA test was used to assess the percentage of variation explained by the treatment along with its statistical significance determined using the Vegan v2.5–3 package. A canonical correspondence analysis (CCA) and redundancy analysis (RDA) were used to investigate the interactions between the soil physicochemical indicators and microbial community structure. The Spearman’s correlation coefficient was used to investigate the relationship between the physicochemical properties of the soil and microbiota abundance, based on the composition distribution of the species. To detect differences between the samples and overall differences and exclude chance data, the significant differences between the two groups were analyzed using a multiple-group sample analysis (eight groups) and two-group analysis of Tb and CK. Multiple-group sample analyzes compared the mean of all samples within each group, while two-group analyzes compared the mean of all samples in the Tb groups with that of the CK groups. The means of the differences in the physicochemical properties of the samples were determined using Duncan’s multiple range test and T-tests in the SPSS Statistics software (version 17.0; IBM Inc., Armonk, NY, United States). The Kruskal–Wallis H test and Wilcoxon rank-sum test of variance were performed using the Majorbio Cloud platform.

## 3 Results

### 3.1 Physicochemical properties of the soil samples

The physicochemical properties of the samples in different groups showed significant differences after the Duncan’s multiple range test (*p* < 0.05) was conducted. The Tb group samples had significantly lower levels of pH, TP, TN, AP, and silt than did the CK group samples in most regions but had significantly higher levels of TK, AN, and sand (Table 2). The T-test yielded comparable findings for the overall analysis of both the CK and Tb groups (Supplementary Table S1).

**Table 2:** Physicochemical properties of *Tricholoma bakamatsutake* shiro soils.

Sample	AK (mg/kg)	AP (mg/kg)	AN (mg/kg)	TK (%)	TP (%)	TN (%)	OM (g/kg)	pH	Sand (%)	Silt (%)	Clay (%)
ASTb	142.6 ± 2.70e	1.133 ± 0.15f	252.10 ± 1.90f	2.41 ± 0.11a	0.069 ± 0.00e	0.402 ± 0.01e	108 ± 2.65d	5.41 ± 0.03d	57.333 ± 0.58b	28.00 ± 1.00 g	14.667 ± 0.58d
ASCK	161.8 ± 3.24c	2.60 ± 0.2e	215.80 ± 3.61 g	2.13 ± 0.03b	0.087 ± 0.00a	0.489 ± 0.00a	116 ± 1.73c	5.66 ± 0.02c	48.00 ± 0.00c	37.00 ± 1.00d	15.00 ± 1.00d
KDTb	127.6 ± 1.76f	3.40 ± 0.17d	322.10 ± 0.95c	1.63 ± 0.05d	0.059 ± 0.00 g	0.412 ± 0.00d	122 ± 1.73b	5.31 ± 0.11e	49.333 ± 0.58c	33.333 ± 0.58e	17.333 ± 0.58c
KDCK	157.8 ± 1.71d	5.50 ± 0.10b	302.20 ± 2.10d	1.33 ± 0.03e	0.072 ± 0.00d	0.452 ± 0.00c	116 ± 2.00c	5.81 ± 0.07b	34.00 ± 1.00e	47.00 ± 1.00b	19.00 ± 0.00b,c
XBTb	36.4 ± 0.28 h	2.70 ± 0.16e	283.10 ± 1.50e	1.39 ± 0.01e	0.065 ± 0.00f	0.348 ± 0.00f	106 ± 1.41d	5.24 ± 0.02e	36.00 ± 1.00d	42.00 ± 1.22c	22.00 ± 1.22a
XBCK	124.6 ± 0.64 g	6.50 ± 0.07a	327.70 ± 0.99b	1.21 ± 0.01f	0.079 ± 0.00b	0.461 ± 0.00b	117 ± 1.58c	6.19 ± 0.02a	35.00 ± 1.41d,e	50.00 ± 1.00a	15.00 ± 1.00d
XYTb	252.6 ± 1.30a	3.30 ± 0.20d	348.20 ± 1.61a	1.78 ± 0.02c	0.054 ± 0.00 h	0.398 ± 0.00e	136 ± 1.73a	6.17 ± 0.01a	60.00 ± 1.00a	30.00 ± 1.00f	10.00 ± 2.00e
XYCK	176.8 ± 2.65b	4.80 ± 0.10c	283.10 ± 2.05e	1.23 ± 0.01f	0.075 ± 0.00c	0.413 ± 0.00d	121 ± 1.00b	5.04 ± 0.03f	30.00 ± 1.00f	50.00 ± 1.00a	20.00 ± 0.00b

Values are presented as the means of all replicates. Means in a column followed by different letters are significantly different at *p* ≤ 0.05, according to the Duncan’s multiple range test.

### 3.2 Diversity of the microbial community

The raw data results from MiSeq for the fungal and bacterial communities are presented in Supplementary Tables S2, S3. Sample sequences were drawn flat according to the minimum number of sample sequences before the analysis. The fungal and bacterial coverage indices were 99.57 to 99.93% and 96.70 to 98.76%, respectively. This indicated that most of the fungal and bacterial taxa were detected in the soil samples, and the sequencing results correctly depicted the microorganisms in the samples.

For the fungal community, 47 fungal OTUs were shared between the Tb and CK groups (Supplementary Figure S1A). The CK groups had more unique OTUs than the Tb groups. The Tb and CK groups were analyzed separately (Supplementary Figure S2). The Tb groups had 63 shared OTUs, with XBTb and KDTb having the lowest (57) and highest (168) number of unique OTUs, respectively. The CK groups had 136 shared OTUs, with ASCK and XYCK having the highest (567) and lowest (92) number of unique OTUs, respectively. For the bacterial community, 663 bacterial OTUs were shared between the Tb and CK groups (Supplementary Figure S1B). The Tb groups had more unique OTUs than the CK groups. In the Tb groups, a total of 1,674 OTUs were observed (Supplementary Figure S3). XBTb had the highest number of unique OTUs (536), while KDTb had the lowest (157). In the CK groups, a total of 1,039 OTUs were observed. ASCK had the highest number of unique OTUs (816), while XYCK had the lowest (163).

The Chao and Shannon indices were used to assess the α diversity of the microbial community, with the former reflecting species richness and the latter reflecting species diversity (Supplementary Figure S4). In the fungal community, both indices of the Tb groups exhibited lower values than those of the CK groups, particularly in the Shannon index, which displayed highly significant differences across all regions (*p* < 0.001). Notably, the Xinbin (XB) region demonstrated both the highest (XBCK) and lowest (XBTb) values for these indices. Regarding the bacterial community, both indices of the Tb groups were higher than those of the CK groups; however, a significant difference was observed only in the Kuandian (KD) region (*p* < 0.01). These findings indicate a significant decrease in species richness and diversity in the fungal community of the shiro but an increasing trend in the bacterial community.

### 3.3 Differences in the microbial relative abundance and community structure

**Figure 1:**
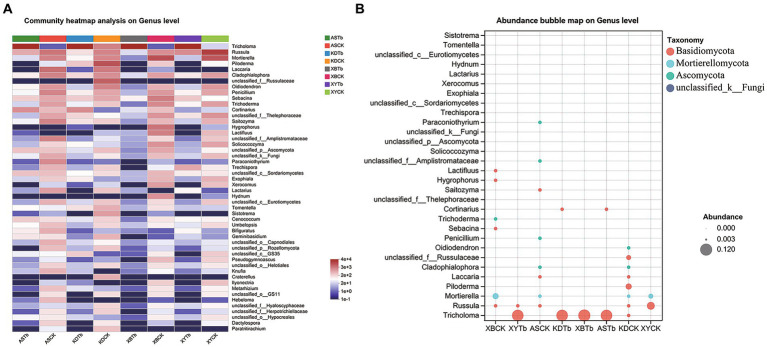
Composition of fungal communities at the genus level in the different groups. **(A)** Heatmap; **(B)** Abundance bubble map. Bubble size and color indicate the abundance and classification of the genus, respectively.

**Figure 2:**
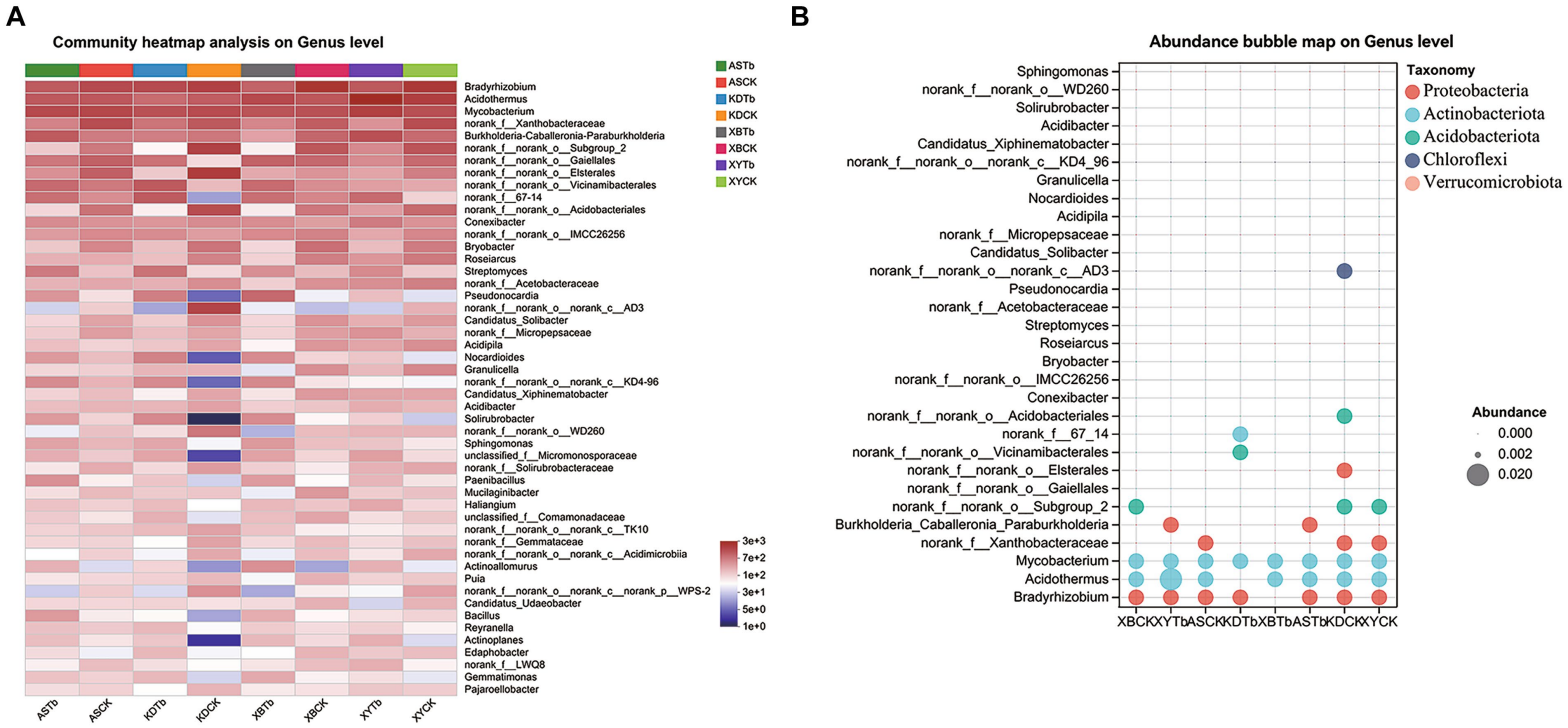
Composition of bacterial communities at the genus level in the different groups. **(A)** Heatmap; **(B)** Abundance bubble map. Bubble size and color indicate the abundance and classification of the genus, respectively.

For the fungal community, Basidiomycota, Ascomycota, and Mortierellomycota were dominant in all the soil groups. Basidiomycota (96.21 to 99.77%) and Ascomycota (56.02 to 83.65%) were the dominant phyla in the Tb and CK groups, respectively. At the genus level, the heatmap and abundance bubble map (Figure 1) indicated that *Tricholoma*, *Russula*, and *Mortierella* were the dominant fungal genera in the different regions. Apart from *Tricholoma*, most genera in the Tb groups were less abundant than those in the CK groups. At the species level, *T. bakamatsutake* was the dominant species in the Tb groups, exhibiting 87.7, 88.0, 99.1, and 89.1% abundance in ASTb, KDTb, XBTb, and XYTb, respectively. For the bacterial community, Actinobacteria, Proteobacteria, and Acidobacteria were dominant in all the soil groups. Proteobacteria and Actinobacteria were the most abundant phyla in the CK and Tb groups, respectively. The genus-level heatmap (Figure 2A) and abundance bubble map (Figure 2B) indicated that the genera *Bradyrhizobium*, *Acidothermus*, *Mycobacterium*, *norank_f__Xanthobacteraceae*, and *Burkholderia-Caballeronia-Paraburkholderia* (BCP) were the dominant genera in both groups.

The Kruskal–Wallis H test (eight groups) and Wilcoxon rank-sum test (CK and Tb) were used to analyze the differences in the abundance of microorganisms between the groups. In the fungal community (Supplementary Figure S5), *T. bakamatsutake* was significantly more abundant in the Tb groups than in the CK groups, whereas almost all the other fungi, such as *Russula* and *Penicillium*, were significantly lower in abundance in the Tb groups than in the CK groups. This suggests that the dominance of Tb has a broad and significant effect on other fungi. However, the dominance of one species did not result in an overwhelming change in the bacterial community. In the Tb groups (Supplementary Figure S6), there was a significant increase in Actinobacteria, such as *norank_f__67–14*, *Streptomyces*, *Pseudonocardia*, *Bradyrhizobium*, *norank_f__Xanthobacteraceae*, *norank_f__norank_o__Elsterales*, and *Nocardioides*. The multigroup analysis showed that BCP was significantly more abundant in ASTb and XYTb than in ASCK and XYCK but less abundant in KDTb and XBTb than in KDCK and XBCK. The abundance of common MHB (Frey-Klett et al., 2007) was further assessed in the analysis (Supplementary Figure S7). The identified MHB, which were significantly more abundant in the Tb groups than in the CK groups, were *Sphingomonas*, *Paenibacillus*, and *Bacillus*.

The NMDS analysis showed differences in the microbiome structures in the CK and Tb groups (Supplementary Figure S8). The fungal communities in the CK and Tb groups also differed. In the CK groups, KDCK was far from the other CK groups, indicating that the structure of the fungal communities of the oak forests in KD differed from that in other areas. The bacterial communities in both groups were generally similarly distant but different from each other.

### 3.4 Correlation between soil physicochemical properties and microorganisms

**Figure 3:**
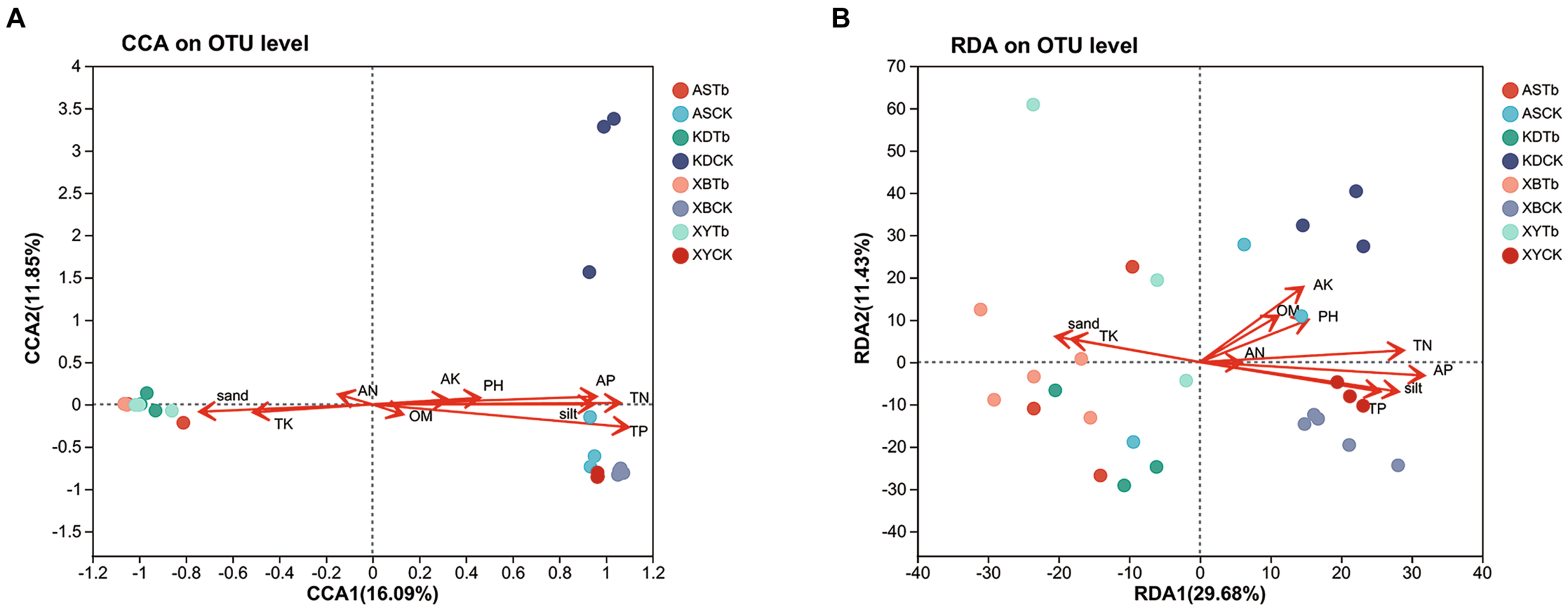
CCA/RDA analysis of the relationship between the microbial communities and soil physicochemical properties. **(A)** Fungal communities; **(B)** Bacterial communities. AP (r^2^ = 0.5386, *p* = 0.001), TP (r^2^ = 0.7456, *p* = 0.001), TN (r^2^ = 0.6587, *p* = 0.001), sand (r^2^ = 0.318, *p* = 0.01), and silt (r^2^ = 0.5227, *p* = 0.001) were significant factors that influenced fungal community structure. AK (r^2^ = 0.2546, *p* = 0.021), AP (r^2^ = 0.6052, *p* = 0.001), TK (r^2^ = 0.203, *p* = 0.049), TP (r^2^ = 0.4041, *p* = 0.001), TN (r^2^ = 0.4976, *p* = 0.001), sand (r^2^ = 0.2562, *p* = 0.025), and silt (r^2^ = 0.4882, *p* = 0.001) were significant factors that influenced the bacterial community structure. CCA/RDA, correspondence analysis/redundancy analysis.

**Figure 4:**
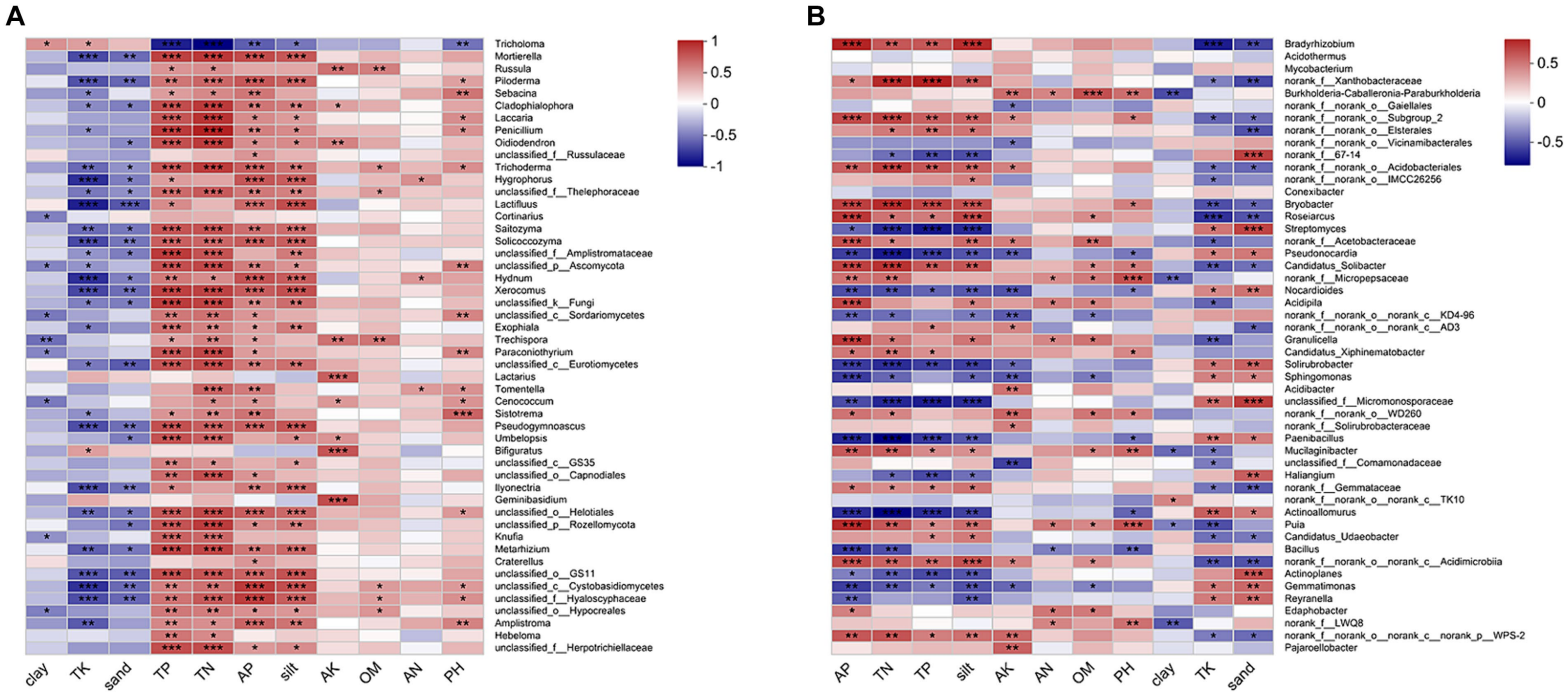
Heatmap of Spearman’s correlation coefficients in the microbial communities and environmental factors. **(A)** Fungal communities; **(B)** Bacterial communities. The correlation coefficient R values are shown in different colors, and the color ranges for different R values are shown in the legend on the right. The squares with a significance mark are the correlation thresholds, where |R| ≥ 0.1. The significance levels are denoted by asterisks: * for 0.01 < *p* ≤ 0.05, ** for 0.001 < *p* ≤ 0.01, and *** for *p* ≤ 0.001.

The CCA/RDA analysis revealed significant impacts of the soil physicochemical properties on the bacterial and fungal communities (Figure 3). The AP, TP, TN, sand, and silt levels exhibited significant correlations with both the fungal and bacterial communities, indicating their pivotal roles in shaping and regulating the microbial community. The Spearman’s analysis revealed significant correlations between the microbial community and physicochemical properties of the soil (Figure 4; Supplementary Table S4). In the fungal community, *T. bakamatsutake* exhibited a positive correlation with the levels of clay, TK, and sand, while displaying a significant negative correlation with the levels of silt, pH, TN, TP, and AP. In the bacterial community, most of the bacteria demonstrated a significant positive correlation with the silt content as well as the TN, TP, and AP levels. Notably, several actinomycetes displayed similar patterns to *T. bakamatsutake* in terms of their associations with the physicochemical properties of the soil. For example, *Streptomyces*, *Pseudonocardia*, *norank_f__67–14*, *Nocardioides*, and *Solirubrobacter* exhibited a positive correlation with the TK and sand levels but a negative association with other physicochemical properties.

### 3.5 Functional predictions of the microorganisms

Functional predictions were made for the bacterial and fungal communities; the number of enzymes and pathways is explained in Supplemental Table S5. The fungal community function was predicted using PICRUSt2 and FunGuid. The results of the enzyme-level analysis facilitated by the Kyoto Encyclopedia of Genes and Genomes (KEGG) indicated that adenosine triphosphatase, glucan 1,4-alpha-glucosidase, and unspecific monooxygenase were the most abundant across the samples. The MetaCyc pathway abundance statistics showed that aerobic respiration I, aerobic respiration II, fatty acid, and beta oxidation were the most abundant metabolic pathways in each sample. The significant differences were analyzed by focusing on the top 50 data points on abundance. The abundances of enzymes (Supplementary Figures S9A,C), such as glucan 1,4-α-glucosidase and carboxylesterase, and metabolic pathways (Supplementary Figures S9B,D), such as aerobic oxidation and fatty acid degradation, were significantly higher in the Tb groups than in the CK groups. Fungal communities were classified using the fungal FunGuid function, with ECM fungi (including ECM and ECM-fungal parasites) being the most abundant in all the groups. An analysis of the significance of these differences (Supplementary Figures S10A,B) showed that ECM-fungal parasites were significantly higher in the Tb groups than in the CK groups, whereas ECM and saprotrophic parasites were significantly lower in the Tb groups than in the CK groups.

Functional predictions of the bacterial communities were performed using PICRUSt2, BugBase, and FAPROTAX. An enzyme-level analysis of the KEGG functions showed that NADH: ubiquinone reductase, DNA-directed DNA polymerase, and DNA helicase were the most abundant across the samples. The MetaCyc pathway abundance statistics showed that aerobic respiration I and pyruvate fermentation of isobutanol were the most abundant pathways in each sample. An analysis of the significant differences showed that the enzymes (Supplementary Figures S11A,C), enoyl-CoA hydratase and long-chain-fatty-acid-CoA ligase, and metabolic pathways (Supplementary Figures S11B,D), fatty acid and beta oxidation I, were significantly more abundant in the Tb groups than in the CK groups. The bacteria were phenotypically predicted using BugBase and analyzed for significant differences (Supplementary Figure S12). Seven of the nine phenotypes differed significantly. The phenotypes that were most frequent in the Tb groups were gram-positive, contained mobile elements, and exhibited stress-tolerance, whereas the phenotypes that were more frequent in the CK groups were facultative, anaerobic, potentially pathogenic, gram-negative, and aerobic. The metabolic and ecological functions of the bacteria were predicted using FAPROTAX software. The results (Supplementary Figure S13) showed that chemoheterotrophy and aerobic chemoheterotrophs were the most abundant in each sample. Chemoheterotrophy, the abundance of aerobic chemoheterotrophs, and aromatic compound degradation were higher in the Tb groups than in the CK groups, whereas nitrogen fixation was lower in the Tb groups than in the CK groups.

## 4 Discussion

### 4.1 Physicochemical properties of *T. bakamatsutake* shiro

The pH range of all the soil samples was 5.04 to 6.19, which falls within the optimal pH range for the growth of ECM fungi, as reported by Yamanaka (2003). This pH range provided a conducive environment for the proliferation of diverse ECM-fungal species. Nitrogen (N) is the most abundant mineral nutrient required by plants (Xie et al., 2022). Due to the continuous accumulation of OM in forests, organic N dominates over inorganic N in soil (Rineau et al., 2015). However, plants cannot absorb organic N directly; thus, they often establish symbiotic relationships with microorganisms to enhance their uptake of inorganic N (Courty et al., 2015). ECM fungi represent a common example, as they convert soil organic N into inorganic forms through the secretion of extracellular enzymes and other mechanisms, thereby increasing the availability of inorganic N for plant assimilation (Rineau et al., 2015; Sebastiana et al., 2022). This may explain why the content of AN was significantly higher in most of the Tb groups than in the CK groups, while TN showed a significant decrease. Phosphorus (P) is a crucial element for plant growth; however, the content of P in all the samples analyzed in this study was found to be low. This occurrence is not uncommon, as P often limits tree productivity in forests worldwide (Plassard and Dell, 2010). Plants engage in a cooperative relationship with ECM fungi to enhance their ability to acquire P, thereby mitigating the P deficiency in the host (Cairney, 2011). This cooperation primarily manifests through ECM-mediated P solubilization and subsequent efficient mineral absorption (Cairney, 2011; Meeds et al., 2021). This could explain the significantly lower levels of TP and AP observed in the Tb groups than in the CK groups in this study. The sand content was significantly higher in the Tb groups than in the CK groups, whereas the silt content was significantly lower in the Tb groups than in the CK groups. These findings are in line with the soil structure of the *T. matsutake* habitat (Ji et al., 2022). Improved air and water permeability in the soil may be necessary for the survival of *T. bakamatsutake*. The relationship between the physicochemical properties and microorganisms was determined using subsequent correlation analyzes.

### 4.2 *T. bakamatsutake* alters microbiome diversity

The fungal communities in the Tb groups exhibited a significant reduction in species richness and diversity, indicating that *T. bakamatsutake* exerted inhibitory and eliminative effects on other fungi, which is consistent with findings from previous studies on ECM fungi (Yu et al., 2021; Shen et al., 2023). Notably, XBTb displayed the lowest fungal diversity in the Tb groups. This could be attributed to the abundant presence of *T. bakamatsutake* fruiting bodies at the XB sampling sites, which would have resulted in a stronger effect of *T. bakamatsutake* on other fungi within the shiro. Conversely, XBCK exhibited the highest fungal diversity among all the samples, suggesting that the soil environment in XB was conducive to the growth of a wide range of fungi. This may also explain why *T. bakamatsutake* was found to be most abundant in the XB area.

For the bacterial communities, the Tb groups had higher bacterial diversity than the CK groups. ECM-fungal roots can support different types of bacteria (Liu et al., 2018), as the expanded mycelial network of ECM fungi provides a habitat for bacterial communities (Miquel Guennoc et al., 2018), and the photosynthetic products obtained from ECM fungi provide nutrition for many bacteria (Read and Perez-Moreno, 2003; van Scholl et al., 2006; Colin et al., 2017). In the present study, *T. bakamatsutake* received most carbohydrates from the plants, and its mycelia expanded in a denser network, attracting various types of bacteria. OM degraded or secreted by *T. bakamatsutake* may cause bacterial aggregation; however, the exact substances need to be confirmed.

### 4.3 Changes and interactions of bacterial communities in shiro

MHB and endophytic bacteria associated with *T. bakamatsutake* were increased in the shiro. MHB can increase mycorrhization by inducing the rapid expansion of fungal mycelia and stimulating the formation of plant lateral roots to increase contact opportunities (Riedlinger et al., 2006; Rigamonte et al., 2010). Mycorrhizal fungi are selective regarding the type of bacteria in their rhizosphere (Izumi and Finlay, 2010), with different mycorrhizal fungi selecting different bacteria based on their requirements. In this study, *Sphingomonas*, *Paenibacillus*, and *Bacillus* were highly abundant and significantly different between the Tb and CK groups. These bacteria, often found in mycorrhizal environments (Bending et al., 2002; Timonen and Hurek, 2006; Nguyen and Bruns, 2015), promote the growth and colonization of ECM fungus mycorrhizae (Requena et al., 1997; Hrynkiewicz et al., 2009; Ji et al., 2022). Species of the genera *Bacillus* and *Paenibacillus* were significantly high in abundance in the *T. matsutake* shiro (Oh et al., 2016). *Sphingomonas* species are endophytic bacteria of ECM fungi, such as in *T. matsutake* cotyledons (Liu et al., 2021), which possess the ability to degrade aromatic compounds and exhibit metabolic activity against toxic pollutants (Yang C. et al., 2022; Yang M. et al., 2022). Thus, several of the abovementioned bacteria may contribute to the growth and development of *T. bakamatsutake* and its root-colonizing mycelia.

*T. bakamatsutake* aggregated plant growth-promoting bacteria (PGPB). Bacterial genera that have been confirmed as PGPB, such as BCP, *Solirubrobacter*, and *Bacillus*, were significantly more abundant in the Tb groups than in the CK groups. In future studies, several genera with high abundances will be explored. BCP are widely distributed in the soil and plant roots and promote plant growth, improve plant resistance, and reduce self-toxicity (Beukes et al., 2017; Luo et al., 2021). In the present study, BCP abundance was higher in both the ASTb and XYTb samples than in the corresponding CK samples. Inter-root secretions are enriched with and degraded by BCP to reduce plant auto-toxicity (Luo et al., 2021). Therefore, the possibility of Tb inter-root secretions, whether enriched with BCP or not, requires further study. In addition to being beneficial to plants, BCP aid the development of *T. matsutake* (Xu, 2021). Actinobacteria are considered special PGPB that promote plant growth and help plants resist pathogenic bacteria in several ways, including through the decomposition of OM, secretion of antibiotics, degradation of pollutants, and secretion of phytohormones (Bhatti et al., 2017; Olanrewaju and Babalola, 2018; Hayat et al., 2021). In the present study, the relative abundance of Actinobacteria was significantly higher in the Tb samples than in the CK samples. The significant increase in Actinobacteria was mainly concentrated in the genera *Streptomyces*, *Pseudonocardia*, *norank_f__67–14*, *Nocardioides*, and *Solirubrobacter*. *Streptomyces*, the most highly anticipated PGPB among actinomycetes, can efficiently colonize inter-root surfaces and promote plant growth through the direct production of phytohormones and decomposition of OM (Verma et al., 2011; Olanrewaju and Babalola, 2018). *Norank_f__67–14* and *Solirubrobacter* species belong to the order Solirubrobacterales, which increases phosphorus flow and inhibits the flow of toxic aluminum and manganese from the soil to the plant, thereby promoting high crop yields (Wang et al., 2022). *Nocardioides* and *Pseudonocardia* are highly metabolically active against toxic pollutants, and their secretions exert antimicrobial activity (Carr et al., 2012; Singha and Pandey, 2021; Yang C. et al., 2022; Yang M. et al., 2022). Therefore, *T. bakamatsutake* aggregates Actinobacteria to degrade soil contaminants, secrete antibiotics to suppress pathogenic bacteria, and provide nutrients for itself, thereby aiding its own growth and development and the oak environment.

Microorganisms interact via metabolites. Actinomycetes in the shiro had the most significant effect on other bacteria, especially gram-negative bacteria. The abundance of *norank_f__norank_o__Elsterales* was lower in the Tb groups than in the CK groups. Elsterales may be associated with plant pathogenicity, soil carbon metabolism, aluminum accumulation, and soil impoverishment (Xu, 2021). This suggests that actinomycetes can suppress microorganisms that are detrimental to *T. bakamatsutake* and oak forests. However, actinomycetes can inhibit beneficial microorganisms, such as BCP. The analysis of the significant differences in the bacterial communities and Spearman’s analysis (Supplementary Figure S14) showed that Actinobacteria, *Pseudonocardia*, *Solirubrobacter*, and *Nocardioides* were significantly negatively correlated with BCP, with significant differences in the abundance of the three genera in XB and KD. Nocardicin A, a metabolite of *Nocardioides*, has a broad-spectrum gram-negative inhibitory effect (Aoki et al., 1976; Nishida et al., 1977; Luo et al., 2013) and is a natural product of *Pseudonocardia* (Carr et al., 2012). Therefore, bacteria that belong to the BCP genera may be more sensitive than other bacteria to the inhibitory effects of these three genera. This explains the low abundance of the BCP in XBTb and KDTb. Other beneficial bacteria, such as *Bryobacter* (Wang et al., 2022) and *Roseiarcus* (Yu et al., 2020), were similarly significantly affected. Despite the inhibitory effects of actinomycetes, beneficial bacteria still prevailed, some of which, including *Bacillus*, were not as negatively affected; therefore, this did not affect the growth and development of *T. bakamatsutake*.

Bacterial competition for ecological niches was considered in this study. *Bradyrhizobium* and *norank_f__Xanthobacteraceae*, which are nitrogen-fixing rhizobia (Rhizobiales) that promote the growth and root development of some plants (Yang C. et al., 2022; Yang M. et al., 2022), were highly abundant in all the samples. Therefore, Rhizobiales are symbionts of *T. bakamatsutake*; however, they were lower in abundance in the Tb groups than in the CK groups, which is notable. In addition to antibiotic antagonism, Rhizobiales compete with other bacteria, such as *Frankia* (Wall, 2000), for nitrogen fixation, ecological niches, and other aspects.

The interactions between bacterial communities are not limited to antagonistic and competitive interactions, and symbiotic interactions were observed in this study. A prime example is the manner in which actinomycetes suppress some bacteria and degrade toxic pollutants to provide nutrients and a good habitat for other bacteria and plants. These effects are not as widespread as the antagonistic effects of actinomycetes; however, they are apparent.

### 4.4 Characteristics of the fungal communities in the soils of shiro and *Q. mongolica* forest

*T. bakamatsutake* dominated its shiro, with the highest relative abundance of 99.1% being in XBTb. Most fungi in the Tb groups were affected by the dominant effect of *T. bakamatsutake*; therefore, the fungi affected by *T. bakamatsutake* were divided into two main groups. The first group comprised other ECM fungi that mainly included *Russula*, *Piloderma*, *Laccaria*, and *Sebacina*. The dominant effect of *T. bakamatsutake* on other ECM fungi is reflected in the competition for ecological niches, such as plant root symbiosis sites and soil nutrients, whereas *T. bakamatsutake* metabolites may have a suppressive effect on other ECM fungi. The second group included reported plant pathogenic fungi, such as *Penicillium* (Li et al., 2010) and *Trichoderma* (Gajera et al., 2015). The dominant effect of *T. bakamatsutake* on pathogenic fungi is reflected in the competition for nutrients and survival space, while actinomycetes enriched by *T. bakamatsutake* inhibit some pathogenic fungi. The decrease in pathogenic fungi promoted the growth and development of *Q. mongolica*. In addition to these two groups of fungi, other common fungi in oak forests were affected by the dominance of *T. bakamatsutake*.

**Figure 5:**
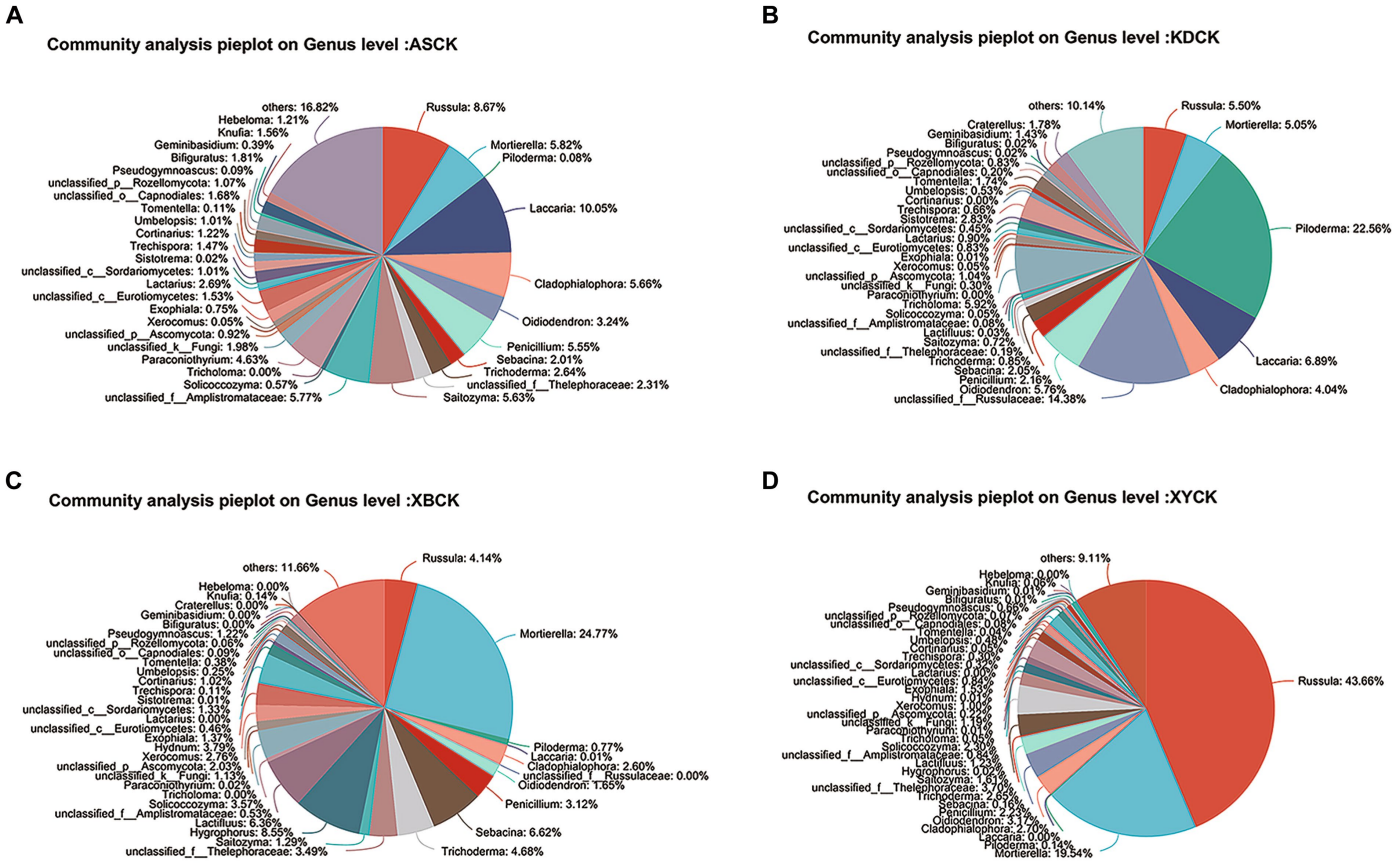
Pie plot of the fungal community composition at the genus level in the *Quercus mongolica* rhizosphere soil (CK) group. **(A)** ASCK; **(B)** KDCK; **(C)** XBCK; **(D)** XYCK.

The fungal communities in the CK groups from the four areas were jointly analyzed (Figure 5). ECM fungi, such as *Russula*, *Piloderm*, and *Lactifluus*, shared a similar habitat with *T. bakamatsutake––*the oak forest. However, these ECM fungi are the main competitors of *T. bakamatsutake*, the most competitive being *Russula*, which is the most abundant among the ECM fungi (Ji et al., 2019). In addition to mycorrhizal fungi, genera, such as *Mortierella*, are common fungi present under oak trees and may contribute to the growth of oak forests (Guo et al., 2022) and indirectly help the formation of symbiotic relationships between oak forests and mycorrhizal fungi.

### 4.5 Potential functions of *T. bakamatsutake* in symbiotic associations

Given the abundance of *T. bakamatsutake* in the Tb groups, the predictive function of the fungal community primarily reflected the functional contribution of *T. bakamatsutake*. The predicted results for the MetaCyc pathways showed a significantly higher abundance of sugar and lipid catabolism-related pathways, such as aerobic respiration I, tricarboxylic acid cycle II, glycolysis III, pentose phosphate pathway, and fatty acid beta oxidation, in the Tb groups compared to those in the CK groups. As a member of ECM fungi, *T. bakamatsutake* mainly obtains nutrients from the host plant sugars and fats for its own nutritional development (Nehls et al., 2010). Therefore, *T. bakamatsutake*’s function is related to the catabolism of sugars and fats. Notably, the glyoxylate cycle was significantly more abundant in the Tb groups than in the CK groups. This suggests that some sugars in *T. bakamatsutake* may not only come directly from the host plant but also be derived from sugars formed by acetyl CoA via the glyoxylate cycle and other pathways. Additionally, octane oxidation was significantly more abundant in the Tb groups than in the CK groups. The application of microbial degradation of aromatic hydrocarbons is of great value; however, studies on the degradation of aromatic compounds by mycorrhizal fungi are limited. Our results indicate that *T. bakamatsutake* may warrant further research in this regard.

The abundance of enzymes was predicted using PICRUSt2. The analysis subsequently focused on enzymes exhibiting higher abundance and greater variation. The glucan 1,4-alpha-glucosidase was the most abundant in the Tb groups and primarily acts on the 1,4-alpha glycosidic bond found in starch. The glucan 1,4-alpha-glucosidase may therefore serve as the pivotal enzyme employed by *T. bakamatsutake* to degrade plant photosynthetic products. Carboxylesterase was significantly more abundant in the Tb groups than in the CK groups and can hydrolyze tannins and corneum from the cell wall. The corneum is a hydroxy fatty acid mixed with wax on the outside of the cell wall to form the cuticle (Samuels et al., 2008; Arya et al., 2021). ECM fungi closely adhere to host roots and secrete cell wall-degrading enzymes to remodel the cell wall, forming a Hartig net that expands the interface for nutrient exchange with the plant (Yu and Yuan, 2023). The plant cuticle is a barrier against microbial infestation (Yeats and Rose, 2013; Ziv et al., 2018), suggesting that carboxylesterases from *T. bakamatsutake* may play a role in their association with host plant root cells. The abundance of chitinase was significantly higher in the Tb groups than in the CK groups and is often observed in ECM fungi, as chitinase degrades the fungal cell wall and releases spores. The abundance of gibberellin 2-beta-dioxygenase, an enzyme involved in gibberellin anabolism, was also increased in the Tb groups compared to that in the CK groups. This may play a facilitative and regulatory role for plant hosts and in the growth of *T. bakamatsutake*. Choline dehydrogenase can oxidize choline, which is subsequently oxidized by the enzyme betaine-aldehyde dehydrogenase to form betaine (Zhang et al., 2020); however, the latter was found to be less abundant in the Tb groups than in the CK groups. Additionally, enzymes such as tripeptidyl-peptidase I and non-specific monooxygenase were significantly more abundant in the Tb groups than in the CK groups, which could lead to interesting findings in future studies.

### 4.6 Prediction of bacterial functions in shiro soils

The bacterial community functions were predicted using PICRUSt2. The Tb groups showed a significantly higher abundance of enzymes involved in fatty acid and sugar catabolism than did the CK groups. The enzymes included enoyl-CoA hydratase, long-chain-fatty-acid-CoA ligase, pyruvate dehydrogenase, and dihydrolipoyl dehydrogenase (Martin et al., 1988; Wang et al., 2002; da Silva Coelho et al., 2010). The abundance of metabolic pathways, such as fatty acid beta oxidation I, was also significantly high in the Tb groups compared to that in the CK groups. Hence, the Tb group soils may have been enriched with bacteria related to fatty acid and sugar catabolism. Additionally, the abundance of betaine-aldehyde dehydrogenase at the enzyme level was significantly higher in the Tb groups than in the CK groups, suggesting that in the Tb groups, the bacterial community may have filled the gap in the fungal community with a lower abundance of this enzyme. Perhaps a collaborative effect between the fungi and bacteria exists for improved betaine formation.

The phenotypes of the bacterial communities were analyzed using data from BugBase. The main factor contributing to the significantly different BugBase phenotypes was the aggregation of actinomycetes in Tb. Actinomycetes had a stronger inhibitory effect on gram-negative bacteria, resulting in a higher proportion of gram-positive and lower proportion of gram-negative bacteria in the Tb groups compared to those in the CK groups (Aoki et al., 1976). The significantly high stress-tolerance phenotype in the Tb groups may have been related to antibiotic stress resistance, as fewer non-resistant bacteria were inhibited in Tb. It is possible that the bacterial communities in the Tb groups exhibited high stress tolerance. Additionally, there was a lower relative abundance of potentially pathogenic bacteria, including some plant pathogenic bacteria, in the Tb groups compared to that in the CK groups. Therefore, both plants and *T. bakamatsutake* itself had a low probability of being attacked by pathogenic bacteria in Tb soils.

The functional annotation of the bacterial communities was performed using FAPROTAX. Chemoheterotrophy and aerobic chemoheterotrophs were more abundant in the Tb groups compared to those in the CK groups. The carbon and energy sources of chemoheterotrophic microorganisms originate from the oxidative decomposition of OM (Zhou, 2020). Saprophytic bacteria were the most abundant among the chemoheterotrophic bacteria. In the Tb groups, *T. bakamatsutake* dominated and significantly reduced the abundance of saprophytic fungi. However, *T. bakamatsutake* had limited capacity and efficiency in decomposing OM (Shah et al., 2016); therefore, an increase in saprophytic bacteria was needed to compensate for the scope and efficiency of saprophytic functions in the Tb groups. Nitrogen fixation was significantly reduced in Tb, possibly due to antagonistic effects within the bacterial community and competition for ecological niches, resulting in a reduction in nitrogen-fixing bacteria, such as *Bradyrhizobium*. The degradation of aromatic compounds was higher in the Tb groups than in the CK groups. Aromatic compounds can cause serious environmental pollution (Seo et al., 2009), and the significantly high abundance of aromatic compound-degrading bacteria in the Tb groups was mainly from the several aforementioned actinomycetes (Ren, 2017). Plant pathogens were significantly less abundant in the Tb groups than in the CK groups, suggesting that some plant pathogenic bacteria were inhibited by Tb.

## 5 Conclusion

**Figure 6:**
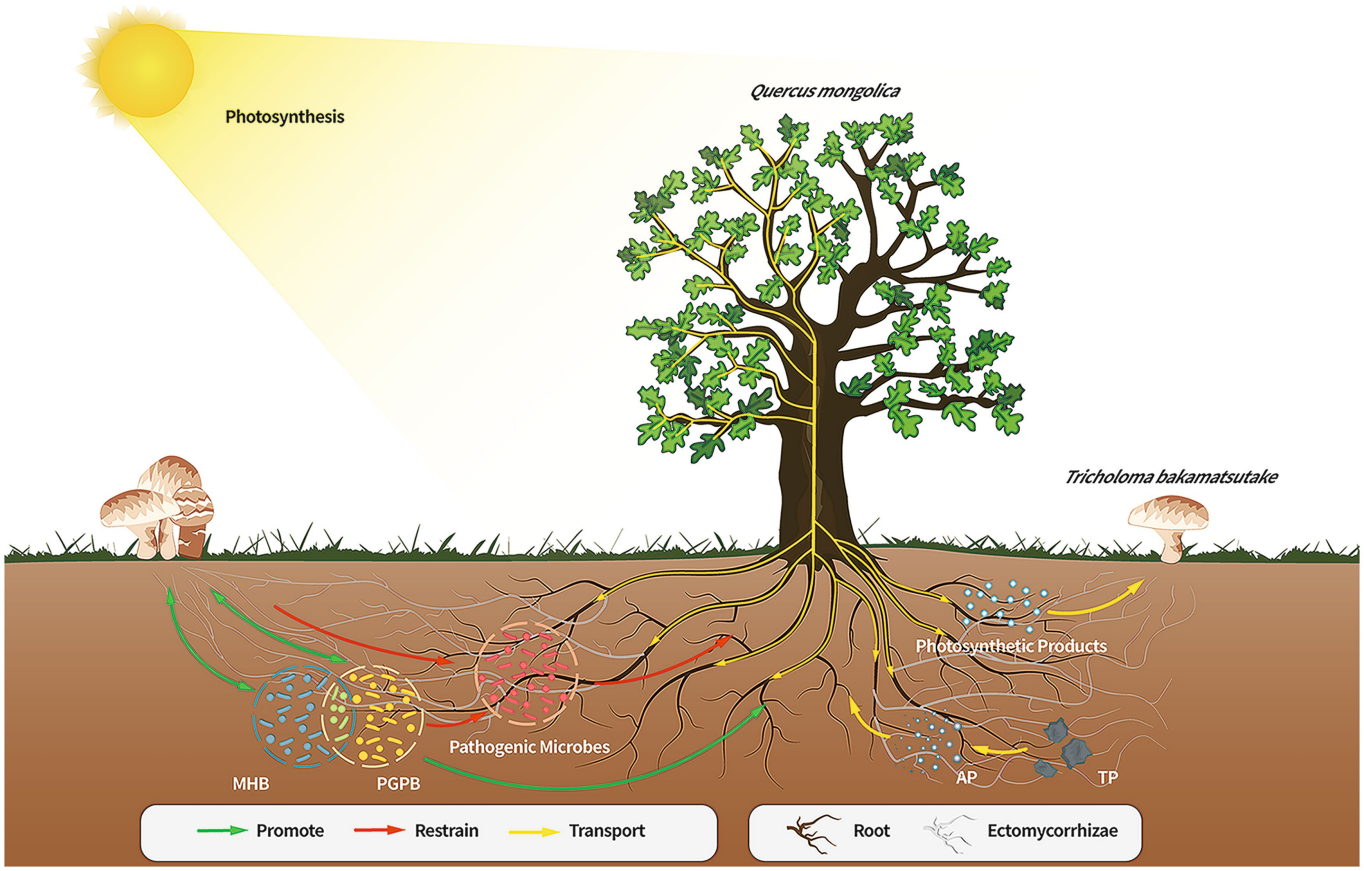
Direct or indirect effects of the members of the *Quercus mongolica*-*Tricholoma bakamatsutake*-associated bacteria symbiosis. The soil of the *Q. mongolica* forest provides a soil environment that contributes to the widespread presence of ECM fungi. However, the composition of the soil affects the colonization of different ECM species, and *T. bakamatsutake* may prefer to colonize environments with high sand and low silt levels owing to good aeration, permeability, and poor water storage; hence, the competition for *T. bakamatsutake* is low in this soil. Once *T. bakamatsutake* colonizes the roots of the oak tree, it receives photosynthetic products from the oak tree through its mycorrhizal roots and transports active soil material to the oak tree. As *T. bakamatsutake* grows, its dominance suppresses other fungi and aggregates its companion bacteria, which are beneficial to its growth and development and those of *Q. mongolica*, whereas *Q. mongolica* and *T. bakamatsutake* provide nutrients to these companion bacteria; ECM, ectomycorrhizal.

In this study, the diversity, microbial populations, and structure of the microbiome in the soils of the Tb and CK groups were compared. Compared to the CK groups, the Tb groups exhibited a clear dominance of *T. bakamatsutake*, resulting in significantly reduced species diversity and richness of other fungal species. On the contrary, the bacterial diversity increased and bacterial community structure was changed in the Tb groups compared to those of the CK groups. There were more abundant PGPB and MHB in the Tb groups than in the CK groups. Abundant MHB (*Sphingomonas*, *Paenibacillus*, and *Bacillus*) promotes fungal hyphal growth and mycorrhizal formation. PGPB are capable of producing plant growth regulators, promoting plant growth, and improving resistance to drought and salt tolerance. The results showed that *T. bakamatsutake* not only directly provided water and minerals to *Q. mongolica* but also promoted the growth of the host by regulating the soil microbial community structure (Figure 6). This study is important for constructing a healthy soil microbial community structure and cultivating *T. bakamatsutake*-*Q. mongolica* mycorrhizal seedlings. In future studies, the symbiotic relationship between *T. bakamatsutake* and *Q. mongolica* can be further examined at the metabolite level, using metabonomics to improve the feasibility of *T. bakamatsutake* artificial cultivation.

## Data availability statement

The datasets presented in this study can be found in online repositories. The names of the repository/repositories and accession number(s) can be found in the article/Supplementary material.

## Author contributions

HG: Writing – review & editing, Writing – original draft, Validation, Supervision, Project administration, Funding acquisition, Formal analysis, Data curation, Conceptualization. WL: Writing – review & editing, Writing – original draft, Visualization, Software, Methodology, Investigation, Formal analysis, Data curation. YX: Writing – original draft, Resources, Investigation. ZW: Writing – original draft, Resources, Investigation. CH: Writing – original draft, Resources, Investigation. JY: Writing – original draft, Validation, Resources. ZY: Writing – original draft, Resources, Investigation. JZ: Writing – original draft, Visualization, Software. XY: Writing – review & editing, Writing – original draft, Validation, Supervision, Resources, Project administration, Funding acquisition, Formal analysis, Data curation, Conceptualization. LS: Writing – review & editing, Writing – original draft, Validation, Supervision, Project administration, Methodology, Conceptualization.

## Footnotes

^1^ https://github.com/picrust/picrust2/

^2^ http://bugbase.cs.umn.edu

^3^ http://www.funguild.org/

^4^ https://cloud.majorbio.com

^5^ http://www.mothur.org

## Glossary

AK: Available potassium
AN: Available nitrogen
AP: Available phosphorus
AS: Anshan
BCP: *Burkholderia-Caballeronia-Paraburkholderia*
CCA: Canonical correspondence analysis
CK: *Q. mongolica* rhizosphere soil
ECM: Ectomycorrhizal
KD: Kuandian
KEGG: Kyoto Encyclopedia of Genes and Genomes
MHB: Mycorrhization helper bacteria
N: Nitrogen
NMDS: Non-metric multidimensional scaling
OM: Organic matter
OTUs: Operational taxonomic units
P: Phosphorus
PCR: Polymerase chain reaction
PGPB: Plant growth-promoting bacteria
PICRUSt2: Phylogenetic Investigation of Communities by Reconstruction of Unobserved States
RDA: Redundancy analysis
rRNA: Ribosomal RNA
Tb: *T. bakamatsutake* shiro soil
TK: Total potassium
TN: Total nitrogen
TP: Total phosphorus
XB: Xinbin
XY: Xiuyan
