# Supplementary material for: Soil microbiome of shiro reveals the symbiotic relationship between Tricholoma bakamatsutake and Quercus mongolica
Source: Front Microbiol. 2024 Mar 27;15:1361117. doi: 10.3389/fmicb.2024.1361117 (PMC11004381; doi:10.3389/fmicb.2024.1361117)
Supplement: Supplementary file 2 [file Table_2.DOCX]

Supplementary Material

1. **Supplementary Figures and Tables**
   1. **Supplementary Figures**

**Supplementary Figure 1.** Venn diagram of OTU number of the different groups. (a) Fungal communities; (b) Bacterial communities.

**Supplementary Figure 2.** UpSet Venn diagram of fungal OTU number in the different groups. (a) Tb groups; (b) CK groups. Set Size (top bar) is a count of the number of OTUs in each group itself. Intersection Size (bottom bar) is a count of the number of OTUs for each group after taking the intersection, with the individual dots below indicating OTUs specific to one group and the line between the dots indicating OTUs for the intersection of different groups. OTU, operational taxonomic unit; Tb, *Tricholoma bakamatsutake* shiro soils; CK, *Quercus mongolica* rhizosphere soils.

**Supplementary Figure 3.** UpSet Venn diagram of bacterial OTU number in the different groups. (a) Tb groups; (b) CK groups.

**Supplementary Figure 4.** Alpha-diversity of bacterial and fungal communities of the different groups. (a) fungal Shannon; (b) fungal Chao; (c) bacterial Shannon; (d) bacterial Chao. Significant difference analysis of diversity indices by Kruskal-Wallis H test: *, P < 0.05; **, P < 0.01; ***, P < 0.001.

**Supplementary Figure 5.** Significance analysis of genus-level differences in fungal communities. (a) Kruskal-Wallis H test; (b) Wilcoxon rank-sum test.

**Supplementary Figure 6.** Significance analysis of genus-level differences in bacterial communities. (a) Kruskal-Wallis H test; (b) Wilcoxon rank-sum test.

**Supplementary Figure 7.** Significance analysis of the differences in the abundance of MHB in different groups. (a) Kruskal-Wallis H test; (b) Wilcoxon rank-sum test. MHB, mycorrhization helper bacteria.

**Supplementary Figure 8.**  Microbial community structure was assessed at the OTU level using NMDS based on the Bray-Curtis. (a) Fungal communities; (b) Bacterial communities.

**Supplementary Figure 9.** Significant differences in the function of fungal communities predicted by PICRUSt2. The Kruskal-Wallis H test for (a) the enzyme level of KEGG function and (b) the MetaCyc pathway; The Wilcoxon rank-sum test for (c) the enzyme level of KEGG function and (d) the MetaCyc pathway. *, P < 0.05; **, P <***, P < 0.001.

**Supplementary Figure 10.** Significant differences in the function of fungal communities predicted by Funguid. (a) Kruskal-Wallis H test; (b) Wilcoxon rank-sum test. *, *P* < 0.05; **, *P* < 0.01; ***, *P* < 0.001.

**Supplementary Figure 11.** Significant differences in the function of bacterial communities predicted by PICRUSt2. The Kruskal-Wallis H test for (a) the enzyme level of KEGG function and (b) the MetaCyc pathway; The Wilcoxon rank-sum test for (c) the enzyme level of KEGG function and (d) the MetaCyc pathway. *, *P* < 0.05; **, *P* < 0.01; ***, *P* < 0.001.

**Supplementary Figure 12.** Significant analysis of the variance of BugBase phenotype predictions for bacterial communities. (a) Kruskal-Wallis H test; (b) Wilcoxon rank-sum test. *, P < 0.05; **, P < 0.01; ***, P < 0.001.

**Supplementary Figure 13.** Functional prediction of FAPROTAX in bacterial communities. (a) Kruskal-Wallis H test; (b) Wilcoxon rank-sum test. *, *P* < 0.05; **, *P* < 0.01; ***, *P* < 0.001.

**Supplementary Figure 14.** Spearman’s analysis of the correlation between bacteria within bacterial community.

- 1. **Supplementary Tables**

**Table S1** Paired samples t-test showed significant differences in soil physicochemical properties between two groups when Sig.(2-tailed) < 0.05.

| Paired Samples | Paired Differences | | | | |  |  |  |
| --- | --- | --- | --- | --- | --- | --- | --- | --- |
|  | Mean | Std. Deviation | Std. Error  Mean | Lower | Upper | t | df | Sig.(2-tailed) |
| TbAK - CKAK | -15.45 | 61.46568 | 17.74361 | -54.50343 | 23.60343 | -0.871 | 11 | 0.403 |
| TbAP - CKAP | -2.21667 | 1.00076 | 0.28889 | -2.85252 | -1.58082 | -7.673 | 11 | 0 |
| TbAN - CKAN | 19.175 | 42.03302 | 12.13389 | -7.53151 | 45.88151 | 1.58 | 11 | 0.142 |
| TbTK - CKTK | 0.3275 | 0.14511 | 0.04189 | 0.2353 | 0.4197 | 7.818 | 11 | 0 |
| TbTP - CKTP | -0.01668 | 0.00351 | 0.00101 | -0.0189 | -0.01445 | -16.47 | 11 | 0 |
| TbTN - CKTN | -0.06383 | 0.04045 | 0.01168 | -0.08953 | -0.03813 | -5.467 | 11 | 0 |
| TbOM - CKOM | 0.5 | 11.18847 | 3.22983 | -6.60881 | 7.60881 | 0.155 | 11 | 0.88 |
| TbPH - CKPH | -0.14417 | 0.81431 | 0.23507 | -0.66155 | 0.37322 | -0.613 | 11 | 0.552 |
| TbSand - CKSand | 13.91667 | 11.10658 | 3.20619 | 6.85988 | 20.97345 | 4.341 | 11 | 0.001 |
| TbSilt - CKSilt | -12.66667 | 5.12274 | 1.47881 | -15.9215 | -9.41184 | -8.565 | 11 | 0 |
| TbClay - CKClay | -1.25 | 6.5661 | 1.89547 | -5.4219 | 2.9219 | -0.659 | 11 | 0.523 |

**Table S2** The sequencing data of fungal communities were obtained from the MiSeq platform.

| Sample\Info | Sequence number | Base number | Mean sequence length |
| --- | --- | --- | --- |
| ASTb1 | 61995 | 16521899 | 266.503734 |
| ASTb2 | 51801 | 14235631 | 274.813826 |
| ASTb3 | 47952 | 13178171 | 274.820049 |
| ASCK1 | 41661 | 10215616 | 245.208132 |
| ASCK2 | 51132 | 13435857 | 262.768071 |
| ASCK3 | 45214 | 12443248 | 275.207856 |
| KDCK1 | 39938 | 11368919 | 284.664205 |
| KDCK2 | 42919 | 11910153 | 277.503041 |
| KDCK3 | 43130 | 11256521 | 260.990517 |
| KDTb1 | 48840 | 13197073 | 270.21034 |
| KDTb2 | 48987 | 13417139 | 273.891828 |
| KDTb3 | 48374 | 13134510 | 271.520031 |
| XBTb1 | 51260 | 14084642 | 274.76867 |
| XBTb2 | 58386 | 16022352 | 274.421128 |
| XBTb3 | 62404 | 17152741 | 274.86605 |
| XBTb4 | 50722 | 13946888 | 274.967233 |
| XBTb5 | 51287 | 14099934 | 274.922183 |
| XBCK1 | 46674 | 11226112 | 240.521747 |
| XBCK2 | 67132 | 16266964 | 242.313114 |
| XBCK3 | 47701 | 11614281 | 243.48087 |
| XBCK4 | 44435 | 10921870 | 245.794306 |
| XBCK5 | 43384 | 10677745 | 246.121727 |
| XYTb1 | 58271 | 15952680 | 273.767054 |
| XYTb2 | 55116 | 15138353 | 274.663492 |
| XYTb3 | 59495 | 16346189 | 274.748954 |
| XYCK1 | 50483 | 12987778 | 257.270329 |
| XYCK2 | 44973 | 11531012 | 256.39855 |
| XYCK3 | 61622 | 15830646 | 256.899257 |

**Table S3** The sequencing data of bacterial communities were obtained from the MiSeq platform.

| Sample\Info | Sequence number | Base number | Mean sequence length |
| --- | --- | --- | --- |
| ASTb1 | 58645 | 24314547 | 414.605627 |
| ASTb2 | 39862 | 16553664 | 415.274296 |
| ASTb3 | 43812 | 18226896 | 416.025199 |
| ASCK1 | 44382 | 18494247 | 416.706029 |
| ASCK2 | 44448 | 18348447 | 412.807033 |
| ASCK3 | 39334 | 16257640 | 413.322825 |
| KDCK1 | 46256 | 19042652 | 411.679609 |
| KDCK2 | 41817 | 17222147 | 411.845589 |
| KDCK3 | 40555 | 16707753 | 411.977635 |
| KDTb1 | 52141 | 21595209 | 414.169444 |
| KDTb2 | 56714 | 23543300 | 415.12325 |
| KDTb3 | 59692 | 24797466 | 415.423608 |
| XBTb1 | 38310 | 15925766 | 415.707805 |
| XBTb2 | 57740 | 23977469 | 415.266176 |
| XBTb3 | 40949 | 16913282 | 413.032846 |
| XBTb4 | 46391 | 19267651 | 415.331659 |
| XBTb5 | 40236 | 16719238 | 415.529327 |
| XBCK1 | 42579 | 17632408 | 414.11043 |
| XBCK2 | 45752 | 18942462 | 414.024786 |
| XBCK3 | 53429 | 22094461 | 413.529375 |
| XBCK4 | 56904 | 23564855 | 414.115967 |
| XBCK5 | 47081 | 19493834 | 414.048852 |
| XYTb1 | 49122 | 20338899 | 414.048675 |
| XYTb2 | 46899 | 19440618 | 414.520949 |
| XYTb3 | 48467 | 20056944 | 413.82681 |
| XYCK1 | 46346 | 19132220 | 412.812756 |
| XYCK2 | 44995 | 18586479 | 413.078764 |
| XYCK3 | 43892 | 18125085 | 412.947348 |

**Table S4**  The Spearman’s analysis between the microbial community and the physicochemical properties of the soil.

Kindly see the attachment: Supplementary Table 4.

**Table S5** Table of enzymes and pathways with numbered names.

| Enzyme/Pathway numbers | Description |
| --- | --- |
| 1.1.99.1 | Choline dehydrogenase |
| 1.14.11.13 | Gibberellin 2-beta-dioxygenase |
| 1.14.14.1 | Unspecific monooxygenase |
| 1.2.4.1 | Pyruvate dehydrogenase |
| 1.8.1.4 | Dihydrolipoyl dehydrogenase |
| 2.7.11.1 | Non-specific serine/threonine protein kinase |
| 3.1.1.1 | Carboxylesterase |
| 3.2.1.14 | Chitinase |
| 3.2.1.3 | Glucan 1,4-alpha-glucosidase |
| 3.4.14.9 | Tripeptidyl-peptidase I |
| 4.2.1.17 | Enoyl-CoA hydratase |
| 6.2.1.3 | Long-chain-fatty-acid--CoA ligase |
| ANAGLYCOLYSIS-PWY | Glycolysis III |
| FAO-PWY | Fatty acid and beta-oxidation I |
| GLYOXYLATE-BYPASS | Glyoxylate cycle |
| NONOXIPENT-PWY | Pentose phosphate pathway |
| P221-PWY | Octane oxidation |
| PENTOSE-P-PWY | Pentose phosphate pathway |
| PWY-3781 | Aerobic respiration I |
| PWY-5067 | Glycogen biosynthesis II (from UDP-D-Glucose) |
| PWY-5083 | NAD/NADH phosphorylation and dephosphorylation |
| PWY-5659 | GDP-mannose biosynthesis |
| PWY-5690 | Tricarboxylic acid cycle II (plants and fungi) |
| PWY-5695 | Urate biosynthesis/inosine 5'-phosphate degradation |
| PWY-7007 | Methyl ketone biosynthesis |
| PWY-7094 | Fatty acid salvage |
| PWY-7279 | Aerobic respiration II |
| PWY-7288 | Fatty acid and beta-oxidation |
